# Estimate selection determines the apparent mortality threshold at 13 mL/kg/h

**DOI:** 10.1186/s13054-026-06328-8

**Published:** 2026-09-15

**Authors:** Shuhao Mei, Hailian Yi, Yuyin Han, Boran Dong, Yong Liu, Xiaoyang Gong

**Affiliations:** 1 Department of Rehabilitation Medicine, The First Affiliated Hospital of Dalian Medical University, Dalian, China; 2 College of Health-Preservation and Wellness, Dalian Medical University, Dalian, China

Dear Editor,

Lumlertgul and colleagues reported that very-low-dose continuous renal replacement therapy (CRRT; <13 mL/kg/h) was associated with higher 90-day mortality in two observational studies (reported odds ratio [OR], 1.72; 95% confidence interval [CI], 1.31–2.24; *I*^2^ = 0%; *P* < 0.001) [1]. This finding is highlighted in the Abstract, Results, Discussion, and Conclusion and is used to support the possibility of a minimum safe CRRT dose. We reconstructed the analysis and found that this apparent threshold depends on which of Faria and Libório’s published estimates is selected for pooling.

Faria and Libório compared a conventional Cox model with a marginal structural Cox model because delivered continuous kidney replacement therapy (CKRT) dose and illness severity changed during treatment [2]. For < 13 versus ≥ 13 mL/kg/h, the conventional model treated whole-course mean dose as a fixed exposure and produced a hazard ratio (HR) of 1.70 (95% CI, 1.16–2.49). Their marginal structural model updated exposure and confounders in 3-day blocks and incorporated treatment and censoring weights. In the graphical results reported by Faria and Libório, the 13-mL/kg/h threshold is assigned an HR of 1.08 (95% CI, 0.74–1.58). By contrast, the accompanying Results text lists the estimates for the 13- and 20-mL/kg/h thresholds in the reverse order, implying an HR of 0.87 (95% CI, 0.66–1.14) at 13 mL/kg/h. Neither time-dependent estimate is statistically significant, and Faria and Libório concluded that time-dependent adjustment nullified the baseline association [2].

The review protocol allowed extraction of univariable or multivariable results but did not specify how to choose among multiple adjusted estimates from one study [3]. Preferred Reporting Items for Systematic Reviews and Meta-Analyses (PRISMA) item 10a asks authors to describe the process used to decide which result to select when several compatible results are available [4]. The review’s assessment using the Risk Of Bias In Non-randomized Studies of Interventions (ROBINS-I) tool also rated the Faria and Libório study at serious risk for selection of reported results because it “reported different modelling.” We reproduced the published subgroup result by transforming the study estimates to the log scale and applying the reported random-effects restricted maximum-likelihood (REML) model. Pooling Faria and Libório’s baseline-only HR of 1.70 with Okamoto’s HR of 1.73 (95% CI, 1.19–2.51) [5] gives 1.715 (95% CI, 1.313–2.240), which rounds to the reported 1.72 (1.31–2.24). Both inputs are Cox HRs, but the pooled result is labelled as an OR.

We then treated estimate selection as a sensitivity analysis rather than as a corrected primary model. Substituting the graphically reported marginal structural estimate from Faria and Libório changes the REML result to 1.369 (95% CI, 0.862–2.172; *P* = 0.183), with *I*^2^ increasing to 66.8% and τ^2^ to 0.074. Using the alternative 0.87 estimate implied by their Results text gives 1.212 (95% CI, 0.618–2.376; *P* = 0.576; *I*^2^ = 88.2%). A fixed-effect sensitivity analysis using 1.08 remains nominally significant (1.372; 95% CI, 1.052–1.790; *P* = 0.020), but it does not match the review’s REML framework. Moreover, the protocol stated that results would not be reported using meta-analysis when significant heterogeneity exceeded 50% [3]. Table 1 presents the principal results, and Additional file 1 provides the equations, intermediate values, and reproducible R code.

The two studies also target different treatment contrasts. Okamoto et al. classified patients by whole-course mean delivered dose, dichotomized at the cohort median of 13.2 mL/kg/h [5]. Faria and Libório’s marginal structural model estimated a longitudinal average treatment effect under changing 3-day treatment histories, time-dependent confounding, treatment crossover, and informative censoring [2]. Methodological guidance recommends selecting the adjusted estimate that best addresses confounding and avoiding quantitative synthesis when design features or estimands are materially incompatible [6]. The alternative pooling therefore demonstrates sensitivity to estimate selection; it should not be interpreted as a definitive replacement estimate.

We recommend that the authors state which estimate from Faria and Libório was selected and why, clarify the inconsistent ordering of the 13- and 20-mL/kg/h estimates between that report’s graphical and textual results, describe the pooled measures consistently as HRs, and present 13 mL/kg/h as an estimate-sensitive exploratory observation. These targeted amendments would align the review’s narrative with its underlying evidence, improve reproducibility, and strengthen its methodological credibility and clinical interpretability by distinguishing the broader dose-intensity evidence from the estimate-sensitive 13 mL/kg/h finding.

**Table 1:** Sensitivity of the < 13 versus ≥ 13 mL/kg/h 90-day mortality subgroup to estimate selection

Analysis	Estimate from Faria and Libório	Okamoto estimate	Pooled ratio (95% CI)	*P*	I^2^
Published reconstruction (REML)	1.70 (1.16–2.49), baseline-only Cox	1.73 (1.19–2.51)	1.715 (1.313–2.240)	< 0.001	0%
Estimate-selection sensitivity (REML)	1.08 (0.74–1.58), marginal structural Cox	1.73 (1.19–2.51)	1.369 (0.862–2.172)	0.183	66.8%
Results-text-order sensitivity (REML)	0.87 (0.66–1.14), marginal structural Cox	1.73 (1.19–2.51)	1.212 (0.618–2.376)	0.576	88.2%
Fixed-effect sensitivity	1.08 (0.74–1.58)	1.73 (1.19–2.51)	1.372 (1.052–1.790)	0.020	66.8% observed

All random-effects analyses used REML with Wald z-based confidence intervals. Both study-level inputs are Cox HRs; no conversion to ORs was applied. The fixed-effect row is a sensitivity analysis, and its *I*^2^ value describes the observed study-level heterogeneity

## Supplementary Information

Below is the link to the electronic supplementary material.

Supplementary Material 1: Additional file 1: File name: Additional_file_1.docx. File format: DOCX. Title: Supplementary Methods 1: Reproducible aggregate-data reconstruction and estimate-selection sensitivity analyses. Description: This file contains source estimates, editable equations, intermediate values, sensitivity analyses, an assessment of whether pooling remains appropriate, and reproducible R code

## Data Availability

No datasets were generated or analysed during the current study.

## Abbreviations

CI: Confidence interval
CKRT: Continuous kidney replacement therapy
CRRT: Continuous renal replacement therapy
HR: Hazard ratio
OR: Odds ratio
PRISMA: Preferred Reporting Items for Systematic Reviews and Meta-Analyses
REML: Restricted maximum likelihood
ROBINS-I: Risk Of Bias In Non-randomized Studies of Interventions
