# Supplementary material for: Estimate selection determines the apparent mortality threshold at 13 mL/kg/h
Source: Crit Care. 2026 Sep 15;30:473. doi: 10.1186/s13054-026-06328-8 (PMC13576319; doi:10.1186/s13054-026-06328-8)
Supplement: Supplementary file 1 [file 13054_2026_6328_MOESM1_ESM.docx]

**Additional file 1**

**Supplementary Methods 1: Reproducible aggregate-data reconstruction and estimate-selection sensitivity analyses**

Estimate selection determines the apparent mortality threshold at 13 mL/kg/h

Shuhao Mei, Hailian Yi, Yuyin Han, Boran Dong, Yong Liu, and Xiaoyang Gong

Critical Care | Corresponding author: Xiaoyang Gong; 18098876676@163.com

**Purpose**

This file reconstructs the published <13 versus ≥13 mL/kg/h 90-day mortality subgroup [1] and evaluates how the result changes when Faria and Libório’s baseline-only Cox estimate is replaced with either threshold-specific estimate reported for the marginal structural Cox model [2]. These estimate-selection sensitivity analyses support targeted corrections to the reporting of estimate selection and to the interpretation of the proposed threshold.

In the graphical results reported by Faria and Libório [2], the 13-mL/kg/h threshold is assigned an HR of 1.08 (95% CI, 0.74–1.58), whereas their accompanying Results text lists the estimates for the 13- and 20-mL/kg/h thresholds in the reverse order. Both possible interpretations are therefore analysed using the source estimates in Table S1.

**Table S1. Source estimates used in the reconstruction**

| **Study/model** | **HR** | **Lower CI** | **Upper CI** | **Role in analysis** |
| --- | --- | --- | --- | --- |
| Faria and Libório baseline-only Cox | 1.70 | 1.16 | 2.49 | Whole-course mean dose; baseline covariate adjustment |
| Faria and Libório MSM, graphically reported 13-mL/kg/h estimate | 1.08 | 0.74 | 1.58 | Time-updated exposure, confounders, treatment weights, and censoring weights |
| Faria and Libório Results-text-order sensitivity | 0.87 | 0.66 | 1.14 | Alternative 13-mL/kg/h estimate implied by reversed textual order |
| Okamoto multivariable Cox | 1.73 | 1.19 | 2.51 | Whole-course mean dose dichotomized at 13.2 mL/kg/h |

**Calculation framework**

Each HR and 95% CI was transformed to the log scale. The between-study variance was estimated by REML. Two-sided Wald P values used the standard normal distribution, matching the review’s reported framework. Standardised equations are provided as editable Word equations; the resulting intermediate values are reported in Table S2, and numerical substitutions are described in the calculation steps below.

$$y_{i}=\ln(\mathrm{HR}_{i});\mathrm{SE}_{i}=\frac{\ln(U_{i})-\ln(L_{i})}{2\times1.96}$$

$$v_{i}=\mathrm{SE}_{i}^{2};w_{i}=\frac{1}{v_{i}+\tau^{2}}$$

$$\overset{^}{\mu}=\frac{\Sigma w_{i}y_{i}}{\Sigma w_{i}};\mathrm{CI}=\exp[\overset{^}{\mu}\pm1.96\sqrt{\frac{1}{\Sigma w_{i}}}]$$

$$I^{2}=\max\{0,\frac{Q-\mathrm{df}}{Q}\}\times100\%$$

**Table S2. Intermediate log-scale values**

| **Study/model** | ***y*_i_ = ln(HR_i_)** | **SE_i_** | ***v*_i_** |
| --- | --- | --- | --- |
| Faria and Libório baseline-only Cox | 0.530628 | 0.194867 | 0.037973 |
| Faria and Libório MSM, HR 1.08 | 0.076961 | 0.193506 | 0.037445 |
| Faria and Libório Results-text estimate, HR 0.87 | −0.139262 | 0.139427 | 0.019440 |
| Okamoto Cox | 0.548121 | 0.190394 | 0.036250 |

**Step 1. Reconstruction of the published result**

Using Faria and Libório’s baseline-only HR of 1.70 (95% CI, 1.16–2.49) [2] and Okamoto’s HR of 1.73 (95% CI, 1.19–2.51) [3], the fixed-effect Q statistic is 0.004123 and *I*^2^ = 0%; REML therefore estimates τ^2^ = 0. The normalized weights are 48.84% and 51.16%. The pooled log effect is 0.539578 with SE = 0.136180, giving 1.715 (95% CI, 1.313–2.240; *P* < 0.001). This reproduces the reported 1.72 (1.31–2.24).

**Step 2. Graphically reported estimate-selection sensitivity analysis**

Using the graphically reported marginal structural HR from Faria and Libório of 1.08 (95% CI, 0.74–1.58) with Okamoto’s HR, Q becomes 3.012335, *I*^2^ = 66.803%, and REML τ^2^ = 0.074149. The normalized random-effects weights are 49.73% and 50.27%. The pooled result is 1.369 (95% CI, 0.862–2.172; *P* = 0.183).

**Step 3. Results-text-order sensitivity analysis**

Using the alternative HR from Faria and Libório of 0.87 (95% CI, 0.66–1.14) with Okamoto’s HR gives 1.212 (95% CI, 0.618–2.376; *P* = 0.576), with *I*^2^ = 88.214% and τ^2^ = 0.208403.

**Step 4. Fixed-effect sensitivity analysis**

Setting τ^2^ = 0 for the HR of 1.08 from Faria and Libório and Okamoto HR 1.73 gives 1.372 (95% CI, 1.052–1.790; *P* = 0.020). This calculation is included for transparency but does not follow the review’s reported random-effects framework.

**Assessment of pooling**

The protocol stated: “If significant heterogeneity is present (>50%), we will not report study results using meta-analysis” [4]. The estimate-selection sensitivity analyses yield *I*^2^ values of 66.8% and 88.2%.

The treatment contrasts also differ. Okamoto classified patients using whole-course mean delivered dose split at 13.2 mL/kg/h. Faria and Libório’s marginal structural model estimated a longitudinal average treatment effect under changing 3-day treatment histories, time-dependent confounding, crossover, and informative censoring. The sensitivity analyses therefore quantify dependence on estimate selection; they are not presented as a definitive replacement causal estimate, and their results are summarised in Table S3.

**Table S3. Summary of sensitivity analyses**

| **Scenario** | **Inputs** | **Pooled ratio (95% CI)** | ***P*** | ***I^2^*** | **τ^2^** |
| --- | --- | --- | --- | --- | --- |
| Published reconstruction, REML | Faria and Libório 1.70 + Okamoto 1.73 | 1.715 (1.313–2.240) | <0.001 | 0% | 0 |
| Graphically reported estimate sensitivity, REML | Faria and Libório 1.08 + Okamoto 1.73 | 1.369 (0.862–2.172) | 0.183 | 66.8% | 0.074 |
| Text-order sensitivity, REML | Faria and Libório 0.87 + Okamoto 1.73 | 1.212 (0.618–2.376) | 0.576 | 88.2% | 0.208 |
| Fixed-effect sensitivity | Faria and Libório 1.08 + Okamoto 1.73 | 1.372 (1.052–1.790) | 0.020 | 66.8% observed | 0 by assumption |

**The complete executable R code is provided in Supplementary Box S1.**

**Supplementary Box S1. Reproducible R code**

| **Line** | **R code** |
| --- | --- |
| 1 | library(metafor) |
| 2 |  |
| 3 | make_dat <- function(study, hr, lower, upper) { |
| 4 | data.frame( |
| 5 | study = study, |
| 6 | yi = log(hr), |
| 7 | sei = (log(upper) - log(lower)) / (2 * qnorm(0.975)) |
| 8 | ) |
| 9 | } |
| 10 |  |
| 11 | run_reml <- function(study, hr, lower, upper) { |
| 12 | dat <- make_dat(study, hr, lower, upper) |
| 13 | rma(yi = yi, sei = sei, method = "REML", test = "z", |
| 14 | slab = study, data = dat) |
| 15 | } |
| 16 |  |
| 17 | published <- run_reml( |
| 18 | c("Faria baseline Cox", "Okamoto Cox"), |
| 19 | c(1.70, 1.73), c(1.16, 1.19), c(2.49, 2.51) |
| 20 | ) |
| 21 |  |
| 22 | time_dependent <- run_reml( |
| 23 | c("Faria and Libório MSM", "Okamoto Cox"), |
| 24 | c(1.08, 1.73), c(0.74, 1.19), c(1.58, 2.51) |
| 25 | ) |
| 26 |  |
| 27 | text_order <- run_reml( |
| 28 | c("Faria text-order", "Okamoto Cox"), |
| 29 | c(0.87, 1.73), c(0.66, 1.19), c(1.14, 2.51) |
| 30 | ) |
| 31 |  |
| 32 | fixed_dat <- make_dat( |
| 33 | c("Faria and Libório MSM", "Okamoto Cox"), |
| 34 | c(1.08, 1.73), c(0.74, 1.19), c(1.58, 2.51) |
| 35 | ) |
| 36 | fixed_effect <- rma(yi = yi, sei = sei, method = "FE", test = "z", |
| 37 | slab = study, data = fixed_dat) |
| 38 |  |
| 39 | published |
| 40 | time_dependent |
| 41 | text_order |
| 42 | fixed_effect |

**References**

1. Lumlertgul N, Kusirisin P, Kung JY, Duquette D, Fujii T, Zarbock A, et al. Lower versus standard dose-intensity continuous renal replacement therapy: a systematic review and meta-analysis. Crit Care. 2026;30:232. doi:10.1186/s13054-026-05987-x.

2. Faria MLM, Libório AB. Continuous kidney replacement therapy dosage and mortality in critically ill patients: a retrospective cohort study using marginal structural model. Shock. 2025;63(2):202–9. doi:10.1097/SHK.0000000000002435.

3. Okamoto K, Fukushima H, Kawaguchi M, Tsuruya K. Low-dose continuous kidney replacement therapy and mortality in critically ill patients with acute kidney injury: a retrospective cohort study. Am J Kidney Dis. 2024;84(2):145–153.e1. doi:10.1053/j.ajkd.2024.01.526.

4. Lumlertgul N, Kusirisin P, Kung JY, Duquette D, Fujii T, Zarbock A, et al. Lower versus standard dose-intensity continuous renal replacement therapy: a protocol for a systematic review and meta-analysis. BMJ Open. 2026;16:e112086. doi:10.1136/bmjopen-2025-112086.
